# Coverage of Health Information by Different Sources in Communities: Implication for COVID-19 Epidemic Response

**DOI:** 10.3390/ijerph17103577

**Published:** 2020-05-20

**Authors:** Bach Xuan Tran, Anh Kim Dang, Phong Khanh Thai, Huong Thi Le, Xuan Thanh Thi Le, Toan Thanh Thi Do, Tu Huu Nguyen, Hai Quang Pham, Hai Thanh Phan, Giang Thu Vu, Dung Tri Phung, Son Hong Nghiem, Thu Ha Nguyen, Trung Dinh Tran, Khanh Nam Do, Dat Van Truong, Giap Van Vu, Carl A. Latkin, Roger C.M. Ho, Cyrus S.H. Ho

**Affiliations:** 1 Institute for Preventive Medicine and Public Health, Hanoi Medical University, Hanoi 100000, Vietnam; dangkimanh@hmu.edu.vn (A.K.D.); lethihuong@hmu.edu.vn (H.T.L.); lethithanhxuan@hmu.edu.vn (X.T.T.L.); dothithanhtoan@hmu.edu.vn (T.T.T.D.); donamkhanh@hmu.edu.vn (K.N.D.); 2 Bloomberg School of Public Health, Johns Hopkins University, Baltimore, MD 21205, USA; carl.latkin@jhu.edu; 3 Queensland Alliance for Environmental Health Sciences (QAEHS), The University of Queensland, Brisbane, QLD 4102, Australia; p.thai@uq.edu.au; 4 Vietnam Young Physicians’ Association, Hanoi 100000, Vietnam; huutu85@gmail.com; 5 Institute for Global Health Innovations, Duy Tan University, Da Nang 550000, Vietnam; phamquanghai@duytan.edu.vn (H.Q.P.); phanthanhhai9@duytan.edu.vn (H.T.P.); 6 Faculty of Medicine, Duy Tan University, Da Nang 550000, Vietnam; 7 Center of Excellence in Evidence-Based Medicine, Nguyen Tat Thanh University, Ho Chi Minh 700000,Vietnam; giang.coentt@gmail.com; 8 School of Medicine, Griffith University, Brisbane, QLD 4111, Australia; d.phung@griffith.edu.au; 9 Centre for Applied Health Economics (CAHE), Griffith University, Brisbane, QLD 4111, Australia; s.nghiem@griffith.edu.au; 10 Department of Pharmacy, Dai Nam University, Hanoi 100000, Vietnam; nghthu11@gmail.com; 11 Demography and Medical statistics, Faculty of Public Health, Danang University of Medical Technology and Pharmacy, Da Nang 550000, Vietnam; trandinhtrung@dhktyduocdn.edu.vn; 12 Department of Pharmacy, University of Medicine and Pharmacy at Ho Chi Minh City, Ho Chi Minh 700000, Vietnam; dattv@ump.edu.vn; 13 Department of Internal Medicine, Hanoi Medical University, Hanoi 100000, Vietnam; vuvangiap@hmu.edu.vn; 14 Respiratory Center, Bach Mai Hospital, Hanoi 100000, Vietnam; 15 Department of Psychological Medicine, Yong Loo Lin School of Medicine, National University of Singapore, Singapore 119228, Singapore; pcmrhcm@nus.edu.sg; 16 Institute for Health Innovation and Technology (iHealthtech), National University of Singapore, Singapore 119077, Singapore; 17 Department of Psychological Medicine, National University Hospital, Singapore 119074, Singapore; cyrushosh@gmail.com

**Keywords:** COVID-19, health communication, social networks, Vietnam

## Abstract

Health personnel and community workers are at the front line of the COVID-19 emergency response and need to be equipped with adequate knowledge related to epidemics for an effective response. This study aimed to identify the coverage of COVID-19 health information via different sources accessed by health workers and community workers in Vietnam. A cross-sectional study using a web-based survey was carried out from January to February 2020 in Vietnam. Respondent-driven sampling (RDS) was used for recruiting participants. We utilized the exploratory factor analysis (EFA) to examine the construct validity of the questionnaire. A higher percentage of participants knew about “Clinical and pathogen characteristics of COVID-19”, compared to “Regulations and policies related to COVID-19”. The percentage of participants accessing the information on “Guidelines and policies on prevention and control of COVID-19” was the lowest, especially among medical students. “Mass media and peer-educators” channels had a higher score of accessing COVID-19 information, compared to “Organizations/ agencies/ associations” sources. Participants consumed most of their COVID-19 information via “Internet, online newspapers, social networks”. Our findings indicate an urgency to re-design training programs and communication activities for a more effective dissemination of information related to the COVID-19 epidemic or epidemics in general.

## 1. Introduction

Coronavirus disease (COVID-19) is an emerging respiratory disease caused by a newly discovered coronavirus and was first detected in Wuhan, China in December 2019 [1]. Due to the threatening impact on health, society, and the economy, this infectious disease was declared a pandemic on March 11, 2020 by the World Health Organization (WHO) [2]. The ongoing COVID-19 pandemic has spread very quickly and, after four months, it has infected over 1.4 million people with more than 85,000 deaths in 212 countries and territories [3]. The health security of Vietnam, one of the countries that shares a border with China, is also threatened with 255 infected cases [4]. It urgently requires Vietnam’s preparedness at the national and provincial levels, with outbreak prevention, and coordinated responses to extinguish the epidemic as rapidly as possible. 

To report on the ability of implementation of the International Health Regulations (IHR), the State Party Self-Assessment Annual Reporting Tool (SPAR) was used for States Parties’ annual reporting [5]. According to the SPAR 2018 report, Europe has a higher score of risk communication (66%) than Vietnam (60%), which presents a real-time exchange of information about disease outbreak between experts or officials and people facing the threats, and how to mitigate the effects of those threats [6]. Moreover, amid an unprecedented global health crisis, we have seen myriad information about COVID-19 circulating in communities, such as infodemics of misinformation concerning dwindling supplies of medical equipment, which increase anxiety within the community [7]. This situation indicates the urgent need to assess the coverage of health information in communities, as misleading information may hamper efforts to contain the epidemic.

There is a diversity of information sources in metropolitan areas, such as mass media, the Internet, social networks, and peer interaction. Research on COVID-19 found that the general population in city areas preferred regular updates of health information via the Internet at the outbreak [8] and during the peak of epidemics [9]. However, those communication measures are not popular in rural areas, which may result in difficulties in COVID-19 information accessibility. Therefore, it is essential to assess the primary and useful information sources about the epidemic in order to help people in communities respond and make informed decisions to prevent and control COVID-19.

Health personnel and community workers are at the front line of any outbreak response. They play critical roles in preventing and controlling any epidemic including the current COVID-19 epidemic [10]. Having the most up-to-date knowledge regarding COVID-19 is necessary in controlling the spread of COVID-19 through appropriate treatment for patients, and protection from infection due to frequent contact with patients [11,12,13]. The WHO also highlights the rights and responsibilities of health workers in order to protect occupational safety and health as they are at a high risk of infection and the source of transmission in the community [14]. Moreover, lessons learned from previous outbreaks and epidemics suggest that knowledge and attitudes regarding infectious diseases are related to the level of adherence to all control measures, which may limit the spread of those diseases. 

During the battle against COVID-19, all countries, even those with developed health systems, face a lack of human resources [15] and a threat to their resilience [16]. Thus, maximizing human resources is one of the keys to success. All health personnel, as well as medical students, can contribute to every stage of epidemic prevention [17]. Medical students who have knowledge of surveillance training, detection tests, and prevention measures will be a useful force in an emergency such as the COVID-19 epidemic [17]. Therefore, this study aimed to identify the information sources of COVID-19, and their contents, accessed by health workers and community workers in Vietnam. This result can inform preparedness efforts to help policymakers to deliver best practices in controlling the COVID-19 disease, particularly in Vietnam.

## 2. Materials and Methods 

### 2.1. Study Setting and Participants

A cross-sectional study utilizing web-based research was carried out from January to February 2020 in Vietnam. This study was a sub-analysis of the overall project on COVID-19 epidemic response assessment from January 2020. During this period of time, Vietnam was highly concerned about COVID-19 cases imported from Hubei, Wuhan, China—the core of the epidemic. Participants who met the following inclusion criteria took part in the survey: (1) aged 18 years old or above; (2) living in Vietnam for at least 6 months; (3) agreeing to be involved in the research by accepting the online informed consent agreement. Our study subjects were medical professionals, medical students, and community workers who were the most influential information receivers in supporting communities to eliminate COVID-19. Thus, in order to create changes in the cognition and behaviors of these subjects, risk communication plays an integral role. During epidemics, effective risk communication, which is the real-time exchange of information between community leaders and the people who are at risk, may allow people to understand and adopt protective behaviors [18]. Understanding and approaching risk communication is critical to perform best practices in preparedness (before), decrease the health risk (during), and disaster resilience (after) the COVID-19 pandemic [18,19].

We utilized the formula of the respondent-driven sampling (RDS) technique for calculating the sample size of the study [20]. The expected proportion of people knowing the coronavirus family before the beginning of COVID-19 infection = 18.8% [12], the margin of error = 0.05, the design effect = 3, and the expected sample size was 504 people. To take into consideration participants not answering completely, we added 25% to the sample size to compensate for people giving incomplete answers, and the expected sample size was 630. A total of 604 participants (medical staff, community workers, medical students) were selected for the sample, with a response rate of 95.9%.

The recruitment process was based on core groups of the Vietnam Young Physician Association, Vietnam Youth Federation, and medical universities in Hanoi, Danang, and Ho Chi Minh City. We selected these groups to reflect the diversity of study subjects, including age, gender, and occupation. The core groups had a higher probability of knowing other people with similar background characteristics and who were eligible to take part in the survey. Community workers were all those working in community facilities. The government of Vietnam mobilized all the political systems, agencies, ministries, branches, and unions, especially those working in the communities—the front line of the combating. Therefore, community workers who are working in community facilities are also the priority subjects of our survey.

### 2.2. Measure and Instruments

*Demographic characteristics*, such as gender, age, marital status and living areas were obtained from the participants together with *Work-related characteristics*, which included the administration level, job title, and workplace location.

*COVID-19-related information channels:* Participants self-reported the channels through which they accessed COVID-19 information, such as training programs at universities/workplaces/unions, news from residential areas/religious activities, mass media, or friends/relatives. Each channel was rated from 0 to 10 for the level of access. Moreover, participants were asked to sort the information content they received into three main categories including clinical and pathological characteristics of COVID-19; guidelines and policies on prevention and control of COVID-19; responsibilities of agencies/organizations/individuals regarding prevention and control of COVID-19.

### 2.3. Data Analysis 

STATA 15.0 (StataCorp LP, College Station, TX) was used to analyze the data. We utilized the exploratory factor analysis (EFA) to examine the construct validity of the questionnaire. The principal component analysis (PCA) was used to define factors with a threshold of an eigenvalue of 1.1 by Scree test, where the curve was flattened. An orthogonal varimax rotation with Kaiser normalization was applied for exploring the scale of items to increase the interpretability of study results. The cut-off point for factor loading was defined at a value of 0.4. We measured the internal consistency of each factor by Cronbach’s alpha. 

Descriptive statistics was adopted to examine characteristic data which covered frequency, percent, mean, and standard deviation. Inferential statistics were applied to perform the comparison among three subject groups by T-test or Mann–Whitney test for quantitative variables and by the Fisher-exact test or Chi-square test for qualitative variables. A Tobit multivariable regression model was applied to identify factors associated with each type of channel. Poisson regression models were used to examine factors related to information content. To obtain reduced models, stepwise forward selection strategies were utilized with a log-likelihood ratio test at a p-value of 0.2. Statistical significance was defined at a *p*-value of less than 0.05.

### 2.4. Ethical Consideration

Participants were asked to provide informed consent prior to conducting the survey. Participants can refuse to take part in or withdraw from the survey at any time. All collected data would be coded and secured and only used for research purposes. Ethical consideration of the study was approved by the Scientific Council of Central Vietnam Youth Union Committee (372a QĐ/TWDTN-VNCTN).

## 3. Results

General information about the participants is presented in Table 1. In total, 30% of participants were male. The majority of participants lived in urban areas (86.4%) and were single (87.7%). Nearly two-thirds of participants worked at the grassroots levels (district and commune levels). The mean age was 22.7 (SD = 5.4).

Table 2 shows the results of the EFA model regarding information sources. Two items were defined from the factor analysis, including “Information from organizations/agencies/associations” and “Mass media and peer-educators”. The first item consisted of five dimensions, with Cronbach’s alpha = 0.88. The second item covered five aspects, and the Cronbach’s alpha was also high at 0.85. About 15.2% of participants always accessed COVID-19 information from training programs at colleges/universities, followed by internet/online newspapers/social networks (13.7%) and 11.7% via radio/television. The mean score of the two items were 5.4 (SD = 2.4) and 6.6 (SD = 1.9), respectively. 

Table 3 depicts the construct validity of information content approached by participants. There were two dimensions determined after factor analysis, namely “Clinical and pathogen characteristics” and “Regulations and policies related to COVID-19”. The values of Cronbach’s alpha were 0.75 (three items) and 0.73 (four items), respectively. A high proportion of participants knew about the causes, transmission, and symptoms of COVID-19 (75.7%), followed by the physical health consequences of COVID-19 (66.3%). Only 30% of participants accessed the information regarding “Guidelines and policies on prevention and control of COVID-19”. 

The primary COVID-19 information contents and the frequency of accessing information sources are described in Table 4. Regarding the type of information, the percentage of participants obtained for “Clinical and pathogen characteristics” was higher than that of “Regulations and policies related to COVID-19” (85.3% and 67.8%, respectively). It is important to note that the proportion of participants who obtained information about “Guidelines and policies on prevention and control of COVID-19” was the lowest, at 29.8%. As was reasonably expected, this proportion among medical students was lower than the medical professionals and community workers groups, and the difference was statistically significant (*p* < 0.01) because the latter groups could receive this information directly from their organization. Moreover, for access to information sources, the score of accessing via “Mass media and peer-educators” was higher than the score of approaching via organizations/ agencies/ associations (6.6 (SD = 1.9) and 5.4 (SD = 2.4), respectively). Accessing through "Internet, online newspapers, social networks" achieved the highest score (7.3 (SD = 2.0)).

Table 5 reveals the factors associated with COVID-19 information sources and contents. Participants aged 25 years old and above were less likely to access COVID-19 epidemic information via both primary sources (Organizations/agencies/associations and mass media/peer-educators). Compared to single participants, those who lived with spouse/family were more likely to know about all of the COVID-19-related information, including “Regulations/ policies” and “Clinical/pathogen characteristics”. Living in rural areas was associated with knowing about “Regulations and policies related to COVID-19”. Regarding information sources, accessing via “Mass media and peer-educators” was positively related to obtaining information about both "Regulations and policies related to COVID-19" and “Clinical and pathogen characteristics”. 

## 4. Discussion

This study showed the differences in accessibility of COVID-19 primary information via multiple channels among medical professionals, medical students, and community workers. "Mass media and peer-educators" was accessed more frequently, compared to "Organizations/agencies/associations". "Guidelines and policies on prevention and control of COVID-19" was the least accessed type of information. This means that in the urgent response to the COVID-19 epidemic, it is necessary to promote communication of up-to-date information on regulations and policies of COVID-19 prevention and control through mass media instead of focusing on the communication of the impact of the epidemic.

Regarding the contents, a high percentage of participants knew about "Clinical and pathogen characteristics of COVID-19". This is consistent with a previous study conducted in Ho Chi Minh City (2020) among health workers, which revealed that large proportions of participants were aware of COVID-19 virus transmission, the main symptoms, and prevention measures [21]. Our findings were higher than the result of a previous survey of healthcare workers, which showed that only 39% of participants knew about COVID-19 epidemic transmission dynamics [11]. In our survey, participants reportedly heard about "Regulations and policies related to COVID-19" less than "Clinical and pathogen characteristics". But it is important to note that this survey was conducted at the early stage of the epidemic when Hubei was on lockdown, and the risk of COVID-19 transmission from China and elsewhere to Vietnam had materialized but was still low. [22] At this time, the first COVID-19 case, who was a foreigner from Wuhan, China, was detected in Vietnam on 24th January. The government of Vietnam focused on exploring the causes, transmission pathways, main symptoms, and protection from COVID-19. On 1st February, when the first case of domestic transmission was investigated, the government took the first action to announce the COVID-19 epidemic across the country. The highest measure implemented so far was to lock down the entire Son Loi commune, which had six COVID-19 cases, on 12th February, to limit the spread of coronavirus. When the number of COVID-19 cases exponentially increased, at the end of March, the Prime Minister decided to temporarily suspend entry for all foreigners and pose the regulation of "social isolation" for at least 15 days nationwide. Communication activities on regulations and policies related to COVID-19 prevention have also been strengthened after the Son Loi lockdown and social isolation, compared to the previous period—the time of conducting the study. However, when the government of Vietnam launched the emergency state for epidemics, the whole system had worked efficiently to respond to COVID-19. It was demonstrated by the high level of adherence to social distancing and “social nearly quarantined” at a very early stage [23].

Among the items of "Regulations and policies related to COVID-19", "Guidelines and policies on prevention and control of COVID-19" received the least attention from participants, especially medical students. Because medical students are a vital force in COVID-19 epidemic prevention, it is important to provide them not only with knowledge about the epidemiology of epidemics [17] but also updated healthcare policies related to COVID-19. As a lesson learned from previous epidemics, understanding the government policies and adhering strictly to those regulations plays a core role in effectively responding and controlling the epidemic [13,24,25]. Therefore, communication activities on regulations and policies related to the prevention and control of COVID-19 should be further promoted, in order to create an effective workforce and collaborating mechanism.

Noticeably, "Mass media and peer-eductors" channels had a higher score of accessing COVID-19 information, compared to "Organizations/ agencies/ associations". Moreover, approaching information via "Mass media and peer-eductors" was more likely to obtain information regarding both "Clinical and pathogen characteristics" and "Regulations and policies related to COVID-19", compared to other sources. Mass media is a dominant information source in this information era since it can provide lightning-speed updates on the epidemic, particularly the number of cases and emergency announcements [26]. Online newspapers can aid in the dissemination of COVID-19 epidemic information, while mass media coverage—including television and printed newspapers—can elicit positive behavioral changes [27]. "Internet, online newspapers, social networks" was the information channel that was most frequently accessed. In fact, public health officials often utilize social networks to stay informed on official information released by government authorities as well as monitor the information authenticity posted by official accounts, which is vital in emergency responses [28]. Social networks and other population-based digital platforms can also offer additional data sources to detect disease outbreaks and cases via the public health surveillance systems [29].

Several implications can be drawn from our study. First, the participants in our study are a key force in COVID-19 prevention and control, and they were aware of their limitations in knowledge about COVID-19. Thus, it is urgent to strengthen training programs to enhance their capacity in epidemic response. Second, communication activities regarding regulations and policies of COVID-19 prevention and control should be further promoted in order for individuals to understand and strictly comply with those regulations fully. "Mass media and peer-educators" are useful information channels to convey timely messages about the epidemic to the people. However, this study does not provide information on the awareness and practice of the community. Therefore, to fully understand the capacity of systems and local communities to control COVID-19, household surveys can provide valuable information about the behaviors, attitudes, and practices of the population in each setting, and at different stages of the epidemic. Currently, the sources of COVID-19 information are well organized and conveyed by the WHO and the national healthcare agencies. We have suggested several highly accredited sources of information on COVID-19, which may be helpful for medical professionals and primary healthcare workers in training and staying informed (Table 6).

The strength of our study is the diversity of the respondents’ backgrounds at the different levels of the health system. However, several limitations still exist in the study. The cross-sectional study design may limit the ability to infer causal relationships. Additionally, it was difficult to generalize our findings across the general population. Data were collected by self-reporting, which may lead to recall bias and social desirability. Although we are aware of the limitations about the representability of the sample population, in times of epidemics, it is not always feasible to obtain a nationally representative sample. Meanwhile, the design for rapid evidence to inform critical policies is vital, and we think that this study can provide a useful reference in Vietnam. In addition, behavioral aspects of the communities were not included in this manuscript, which may have indirect effects on medical professionals’ behaviors in their respective workplaces.

## 5. Conclusions

The findings of the study showed the differences in accessibility of COVID-19 information via multiple communication channels among medical professionals, medical students, and community workers. "Regulations and policies related to COVID-19" received the least attention while "Mass media and peer-educators" was the information channel most frequently accessed. This findings provide evidence for urgently formulating/re-designing training programs and communication activities to enhance the capacity of those people in rapidly responding to the COVID-19 epidemic. 

## Figures and Tables

**Table 1:** Demographic characteristics of participants.

	Medical Professional		Medical Students		Community Workers		Total		*p*-Value
	n	%	n	%	n	%	n	%	
**Total**	41	6.8	487	80.6	76	12.6	604	100.0	
**Gender**									
Male	21	51.2	116	23.8	44	58.7	181	30.0	<0.01
Female	20	48.8	371	76.2	31	41.3	422	70.0	
**Living area**									
Urban	32	78.1	424	87.2	64	85.3	520	86.4	0.25
Rural	9	22.0	62	12.8	11	14.7	82	13.6	
**Region**									
Northern	20	50.0	123	25.8	31	44.9	174	29.7	<0.01
Central	6	15.0	18	3.8	14	20.3	38	6.5	
South	14	35.0	336	70.4	24	34.8	374	63.8	
**Marital status**									
Single	21	51.2	483	99.4	25	32.9	529	87.7	<0.10
Married	20	48.8	1	0.2	48	63.2	69	11.4	
Other	0	0.0	2	0.4	3	4.0	5	0.8	
**Administration level of workplace**									
Center	6	14.6	57	12.0	19	26.4	82	14.0	<0.01
Province	20	48.8	54	11.4	34	47.2	108	18.4	
Below province	7	17.1	353	74.5	0	0.0	360	61.3	
College/University	8	19.5	10	2.1	19	26.4	37	6.3	
**Participating in community activities**									
Yes	30	73.2	199	41.0	76	100.0	305	50.6	<0.01
No	11	26.8	287	59.1	0	0.0	298	49.4	
**Age group**									
Under 25	5	13.9	446	98.7	3	4.0	454	80.6	<0.01
25–29	31	86.1	6	1.3	72	96.0	109	19.4	
	**Mean**	**SD**	**Mean**	**SD**	**Mean**	**SD**	**Mean**	**SD**	***p* Value**
**Age**	31.1	8.1	20.5	1.6	32.1	4.7	22.7	5.4	< 0.01

**Table 2:** Channels of accessing information regarding prevention and control of COVID-19.

Items	Always		Information from Agencies ^a^	Mass Media and Peer-Educators
	n	%		
Training programs at colleges/universities	92	15.2		0.59
Internet, online newspapers, social networks	83	13.7		0.91
Radio and television	71	11.7		0.65
Training at workplaces	50	8.3	0.73	
Printed newspapers	49	8.1		0.74
Relatives	47	7.8		0.63
Unions, associations, clubs	40	6.6	0.73	
Information and instructions from communities of residence	38	6.3	0.78	
Friends and neighbors	36	6.0	0.59	
Religious and belief activities	32	5.3	0.88	
**Cronbach’s alpha**			0.88	0.85
**Mean**			5.4	6.6
**SD**			2.4	1.9

**^a^** Agencies include news agencies as well as organizations and associations.

**Table 3:** Contents of information obtained by participants.

Items	n	%	Clinical and Pathogen Characteristics	Regulations and Policies Related to COVID-19
Causes, transmission, symptoms of COVID-19	460	75.7	0.82	
Physical health consequences of COVID-19	403	66.3	0.80	
Treatment and control methods of COVID-19	383	63.0	0.76	
Responsibilities of individuals and communities for the prevention and control of COVID-19	332	54.6		0.65
Socio-economic consequences of COVID-19	194	31.9		0.69
Responsibilities of agencies and organizations for the prevention and control of COVID-19	194	31.9		0.84
Guidelines and policies on prevention and control of COVID-19	182	29.9		0.77
**Cronbach’s alpha**			0.75	0.73

**Table 4:** Contents regarding COVID-19 and frequency of accessing among participants.

Information Domains	Medical Professionals		Medical Students		Community Workers		Total		*p*-Value
	n	%	n	%	n	%	n	%	
**Clinical and pathogen characteristics**	36	87.8	410	84.0	70	92.1	516	85.3	0.16
Causes, transmission, symptoms of COVID-19	34	82.9	365	74.8	59	77.6	458	75.7	0.46
Physical health consequences of COVID-19	28	68.3	318	65.2	55	72.4	401	66.3	0.45
Treatment and control methods of COVID-19	29	70.7	297	60.9	56	73.7	382	63.1	0.06
**Regulations and policies related to COVID-19**	30	73.2	325	66.6	55	72.4	410	67.8	0.45
Responsibilities of individuals and communities for the prevention and control of COVID-19	25	61.0	267	54.7	39	51.3	331	54.7	0.61
Socio-economic consequences of COVID-19	15	36.6	148	30.3	29	38.2	192	31.7	0.31
Responsibilities of agencies and organizations for the prevention and control of COVID-19	16	39.0	145	29.7	31	40.8	192	31.7	0.09
Guidelines and policies on prevention and control of COVID-19	16	39.0	128	26.2	36	47.4	180	29.8	<0.01
**Information sources**	**Mean**	**SD**	**Mean**	**SD**	**Mean**	**SD**	**Mean**	**SD**	***p* Value**
**Organizations/agencies/associations**	4.8	2.1	5.6	2.4	4.6	2.2	5.4	2.4	<0.01
Friends and neighbors	4.9	2.4	6.0	2.5	5.1	2.3	5.8	2.5	<0.01
Training at workplaces	6.3	2.3	5.9	2.9	5.1	3.1	5.8	2.9	0.09
Unions, associations, clubs	4.4	2.8	5.9	2.8	5.2	3.0	5.7	2.8	<0.01
Information and instructions from residential areas	5.0	2.7	5.6	2.8	4.6	2.6	5.5	2.8	<0.01
Religious and belief activities in pagodas and churches	3.7	3.2	4.6	3.3	3.1	2.9	4.3	3.3	<0.01
**Mass media and peer-educators**	6.0	1.6	6.7	2.0	6.3	1.5	6.6	1.9	<0.01
Internet, online newspapers, social networks	7.1	2.0	7.3	2.0	7.3	1.9	7.3	2.0	0.72
Training programs at colleges/ universities	6.3	2.4	7.3	2.2	5.5	2.7	7.0	2.4	<0.01
Radio and television	6.3	2.3	6.3	2.7	6.8	2.1	6.4	2.6	0.46
Printed newspapers	5.0	3.0	6.3	2.6	6.2	2.2	6.2	2.6	<0.01
Relatives	5.4	2.1	6.4	2.4	5.6	2.2	6.2	2.4	<0.01

**Table 5:** Factors associated with COVID-19 information sources and contents.

	Information from Organizations/Agencies/ Associations		Mass Media and Peer-Educators		Regulations and Policies Related to COVID-19		Clinical and Pathogen Characteristics	
	Coef.	95% CI	Coef.	95% CI	OR	95% CI	OR	95% CI
**Gender** (Female vs male)	−0.48 *	−0.98; 0.02			0.90	0.79; 1.02		
**Age group** (25 and above vs Under 25)	−1.52 ***	−2.14; −0.91	−1.31 ***	−1.99; −0.63				
**Marital status** (vs Single)								
Living with a spouse					1.20 **	1.00; 1.44	1.10 ***	1.03; 1.19
Others			1.51	−0.46; 3.49			1.09 **	1.01; 1.17
**Participated in community activities** (Yes vs no)	0.34	−0.13; 0.81						
**Living area** (Rural vs urban)	0.44	−0.18; 1.06			1.22 ***	1.07; 1.40		
**Region** (vs Northern)								
Central					1.17	0.95; 1.43		
South					1.16 *	1.00; 1.34		
**Objects** (Community workers vs medical professionals)			0.78 *	−0.03; 1.59				
**Information sources**								
Organizations/ agencies/ associations					0.97	0.94; 1.01	0.95 ***	0.93; 0.97
Mass media and peer-educators					1.12 ***	1.06; 1.18	1.07 ***	1.04; 1.10

*** *p* < 0.01, ** *p* < 0.05, * *p* < 0.1.

**Table 6:** Official sources of COVID-19-related information internationally and in Vietnam.

National Authorities and Public Health Agencies	Website	Type of Information
Government portal of the Socialist Republic of Vietnam	http://chinhphu.vn/portal/page/portal/English	Official information provided by the Vietnamese Government portal for a quick update on the COVID-19 situation in Vietnam.
Ministry of Health of Vietnam	https://ncov.moh.gov.vn/	The Ministry of Health of Vietnam portal offers information on Severe acute respiratory syndrome coronavirus 2 (COVID-19).
VTV News - Vietnam Television	https://vtv.vn/	The website of Vietnam Television Station provides official and reputable COVID-19 information sources with rapid updates.
The World Health Organization (WHO)	https://www.who.int/emergencies/diseases/novel-coronavirus-2019	The most updated COVID-19 information across the world provided by the World Health Organization (WHO), including the COVID-19 outbreak situation, COVID-19 country missions, and a range of guidelines and documents related to COVID-19 prevention and control.
European Centre for Disease Prevention and Control	https://www.ecdc.europa.eu/en/COVID-19/sources-updated	The website offers official national information resources on COVID-19 in each country, including websites and national helplines
